# Using WhatsApp messenger for health systems research: a scoping review of available literature

**DOI:** 10.1093/heapol/czab024

**Published:** 2021-04-16

**Authors:** Karima Manji, Johanna Hanefeld, Jo Vearey, Helen Walls, Thea de Gruchy

**Affiliations:** Department of Global Health and Development Affiliation, Faculty of Public Health and Policy, London School of Hygiene & Tropical Medicine (LSHTM), 15-17 Tavistock Place, Kings Cross, London WC1H 9SH, UK; Department of Global Health and Development Affiliation, Faculty of Public Health and Policy, London School of Hygiene & Tropical Medicine (LSHTM), 15-17 Tavistock Place, Kings Cross, London WC1H 9SH, UK; The African Centre for Migration & Society (ACMS), University of the Witwatersrand (Wits), Solomon Mahlangu House, Braamfontein Campus East, Private Bag 3, Johannesburg 2000, South Africa; Department of Global Health and Development Affiliation, Faculty of Public Health and Policy, London School of Hygiene & Tropical Medicine (LSHTM), 15-17 Tavistock Place, Kings Cross, London WC1H 9SH, UK; The African Centre for Migration & Society (ACMS), University of the Witwatersrand (Wits), Solomon Mahlangu House, Braamfontein Campus East, Private Bag 3, Johannesburg 2000, South Africa

**Keywords:** Mobile instant messaging, WhatsApp, health systems research, migrant and mobile populations, low- and middle-income countries, Sub-Saharan Africa

## Abstract

Globally, the use of mobile phones for improving access to healthcare and conducting health research has gained traction in recent years as rates of ownership increase, particularly in low- and middle-income countries (LMICs). Mobile instant messaging applications, including WhatsApp Messenger, provide new and affordable opportunities for health research across time and place, potentially addressing the challenges of maintaining contact and participation involved in research with migrant and mobile populations, for example. However, little is known about the opportunities and challenges associated with the use of WhatsApp as a tool for health research. To inform our study, we conducted a scoping review of published health research that uses WhatsApp as a data collection tool. A key reason for focusing on WhatsApp is the ability to retain contact with participants when they cross international borders. Five key public health databases were searched for articles containing the words ‘WhatsApp’ and ‘health research’ in their titles and abstracts. We identified 69 articles, 16 of which met our inclusion criteria for review. We extracted data pertaining to the characteristics of the research. Across the 16 studies—11 of which were based in LMICs—WhatsApp was primarily used in one of two ways. In the eight quantitative studies identified, seven used WhatsApp to send hyperlinks to online surveys. With one exception, the eight studies that employed a qualitative (*n* = 6) or mixed-method (*n* = 2) design analysed the WhatsApp content generated through a WhatsApp-based programmatic intervention. We found a lack of attention paid to research ethics across the studies, which is concerning given the controversies WhatsApp has faced with regard to data protection in relation to end-to-end encryption. We provide recommendations to address these issues for researchers considering using WhatsApp as a data collection tool over time and place.

**KEY MESSAGES**

- WhatsApp Messenger provides new and affordable opportunities for health research across time and place, potentially addressing the challenges of maintaining contact and participation involved in research with migrant and mobile populations, for example.
- However, little is known about the opportunities and challenges associated with the use of WhatsApp as a tool for health research.
- Reviewing the literature reveals that most of the studies using WhatsApp as a data collection tool for health research have been undertaken in low-and-middle-income countries and that WhatsApp was primarily used either to send hyperlinks to online surveys or to analyse the WhatsApp content generated through a WhatsApp-based intervention.
- These studies pay little to no attention to research ethics, which is concerning given the controversies WhatsApp has faced with regard to data protection in relation to end-to-end encryption.
- We provide recommendations to address these issues for researchers considering using WhatsApp as a data collection tool over time and place.

## Introduction

A growing body of literature addresses the role that increased ownership and use of mobile phones can play in improving both access to healthcare and health systems research in low-and middle-income countries (LMICs), specifically in sub-Saharan Africa (Bloomfield *et al.*, 2014; Hampshire *et al.*, 2015; Lee *et al.*, 2017). The sub-Saharan African region is characterized by mixed migration flows and multiple health challenges, including HIV and tuberculosis, that, due to the inequalities experienced in access to healthcare disproportionately affect many groups—including migrants and mobile populations (Vearey et al., 2017; Vearey, 2018). Given the existing structural factors impeding access to healthcare, coupled with high rates of mobile telephone use across the sub-Saharan African region, ‘mobile health’ or ‘mHealth’—broadly defined as the use of mobile phones in health systems (Noordam *et al.*, 2011)—is consistently recognized as having great potential for improving access to healthcare in this context (Bloomfield *et al.*, 2014; Hampshire *et al.*, 2015; Lee *et al.*, 2017). Its application ranges from the use of mobile phones to improve point-of-care data collection, delivery and communication to real-time medication monitoring and adherence support (Bervell and Al-Samarraie, 2019). Such mobile technologies also offer opportunities for health systems research.

The Migration, Gender and Health Systems (MiGHS) project—a collaboration between the Universities of Cape Town and the Witwatersrand, the London School of Hygiene and Tropical Medicine, and the South African National Department of Health (NDoH)—is researching the impact of migration and mobility on the South African public healthcare system. We identified a gap in methodologies that are able to capture ‘real-time’ data about the healthcare-seeking experiences and interactions with healthcare systems that migrant and mobile populations have over time and place.

To this end, we are exploring the use of WhatsApp Messenger (‘WhatsApp’), a Mobile Instant Messaging (MIM) platform, as a tool for conducting longitudinal research on health systems use by migrant and mobile communities in South Africa. We focus on WhatsApp due to the specific opportunities it presents for undertaking health systems research across both time and place with migrant and mobile populations, including those moving within South Africa (internal migrants) and those crossing borders (international migrants, including refugees and asylum seekers). Our decision to focus on WhatsApp is informed by several key observations, including those drawn from existing literature.

Firstly, mobile phones play important and diverse roles in the lives of migrants, both in the Global North and South (Bacishoga *et al.*, 2016; DA Silva Braga, 2016; Frouws *et al.*, 2016; Lim and Pham, 2016; Alencar *et al.*, 2019; Mancini *et al.*, 2019; Mattelart, 2019; Alencar, 2020; Godin and Donà, 2020; Greene, 2020), including in South Africa (Marchetti-Mercer and Swartz, 2020). WhatsApp is a prevalent and affordable platform in South and Southern Africa (Shambare, 2014; Pindayi, 2017; Dahir, 2018).

Secondly, WhatsApp facilitates the collection of ‘real-time’ data over both time and place. This is achieved through two key functions; participants are able to share their location via WhatsApp, capturing experiences as they are happening and WhatsApp enables users to keep the same mobile phone number and/or account should they cross international borders. The ability to retain the same number has long been a feature of WhatsApp, but recent updates mean that if the number associated with a WhatsApp account is changed, contacts are notified of the change. As such, if a research participant changes their number, they would remain contactable by a research team.

Finally, WhatsApp can also interface with online platforms that allow for the automatic administration of surveys through WhatsApp. The latter function, which is unique to WhatsApp, warrants an independent review of the use of WhatsApp as a data collection tool, given its potential for conducting health research.

Whilst WhatsApp has been successfully used in research with migrant and mobile groups (Almenara-Niebla and Ascanio-Sánchez, 2020; Khoso *et al.*, 2020), little is known about the use of WhatsApp in health systems research. To address this gap, we have undertaken a scoping review exploring the use of WhatsApp in health systems research. In doing so, we hope to glean lessons learned on how best to design and implement research using WhatsApp with migrant and mobile communities in South Africa. Given the well-documented sensitivities that can emerge when conducting research with migrant and mobile groups (Duvell *et al.*, 2008; Ahmed *et al.*, 2019), we pay particular attention in our review to the approaches taken to protect participants’ privacy.

After providing an overview of MIM approaches and WhatsApp more specifically, we present the methodology for our scoping review, followed by our findings. We then discuss the implications for health systems research and conclude with recommendations for researchers interested in exploring the use of WhatsApp as a research tool.

### Mobile Instant Messaging and the use of WhatsApp Messenger for health systems research

Many mHealth interventions make use of Mobile Instant Messaging (MIM), a feature which allows smartphone users to connect to the internet to send real-time text messages to individuals or groups at little or no cost (Church and De Oliveira, 2013). The real-time text message feature of MIM provides an easy-to-use tool for data collection: it enables immediate communication between researcher and participant; and offers flexibility regarding place and time of use as participants and investigators do not have to share a geographic location (Kaufmann, 2018b; Kaufmann and Peil, 2019). As a result, research using MIM can be carried out wherever there is internet connectivity, via cell phone networks or Wi-Fi, thus providing new opportunities for research. This is particularly relevant when working with communities, including migrant and mobile populations, that are difficult to reach and/or to maintain contact with over time when using more traditional research methods such as face-to-face interviews and administered surveys (Kaufmann and Peil, 2019).

Globally, WhatsApp Messenger (‘WhatsApp’) has emerged as one of the world’s fastest-growing MIM applications (Endeley, 2018; Fiesler and Hallinan, 2018), and, by February 2020 had 2 billion users in >100 countries (WHATSAPP, 2020). The WhatsApp software offers a plethora of health-related uses, including for optimizing communication and the delivery of health education (Araújo *et al.*, 2019; Lima *et al.*, 2019). It has particularly high penetration rates in India, Indonesia, Malaysia, Brazil and South Africa (Dahir, 2018; Fiesler and Hallinan, 2018). Most recently, WhatsApp has formed part of both South Africa and the World Health Organization’s (WHO) responses to the SARS-CoV-2 pandemic (DEPARTMENT OF HEALTH, S. A., 2020; Farai, 2020). In March 2020, Health Connect was created for the South African National Department of Health (NDoH) by Praekelt.org, building on Praekelt.org’s experience with national mHealth programmes, including the established MomConnect application (Seebregts *et al.*, 2018). The Health Connect software has since been used by the WHO to create their own WHO HealthAlert Covid19 chat service, indicating the opportunities and reach provided by WhatsApp globally (Farai, 2020).

### Methodological and ethical concerns

The use of WhatsApp necessitates consideration of key methodological, practical and ethical questions (Boase, 2013; Tagg *et al.*, 2017; Barbosa and Milan, 2019). For example, there is a need for adequate infrastructure, including reliable access to electricity and the internet, and ownership of smartphones capable of running WhatsApp (Tagg *et al.*, 2017). Gender and other equity-related differences in the use of mobile technology must also be carefully considered (Noordam *et al.*, 2011). For example, for many people in Southern Africa, access to a WhatsApp compatible phone remains restrictively expensive. There is also a growing body of literature, particularly from developing countries, on the significant gender divide in access to mobile phones, with men being far more likely to have access to a device than women (Blumenstock and Eagle, 2010; Zainudeen et al., 2010; Murphy and Priebe, 2011). Some studies reveal the nuanced intersections of mobile phone usage with gender, poverty and other social strata: findings from a study in Rwanda (Blumenstock and Eagle, 2010) indicate that phone owners are wealthier, better educated and predominantly men when compared to the general population. Research using WhatsApp thus has the potential to exacerbate existing inequities, if such considerations are not thoughtfully addressed beforehand.

Ensuring the privacy and confidentiality of participants and data are also critical when engaging with WhatsApp as a research tool, due to ongoing concerns with the application’s security (Kimmel and Kestenbaum, 2014; Kaufmann and Peil, 2019). Although communication on WhatsApp has been encrypted since 2016, allowing data between communicating parties to be secure, this does not stop Facebook—who purchased WhatsApp in 2014—from accessing and using data collected from subscribers, without their affirmative consent (Kimmel and Kestenbaum, 2014). Nor does the encryption technology guarantee privacy from government surveillance for national security purposes (Endeley, 2018). Further, Ganguly (2017) has reported a design feature in WhatsApp that could potentially allow some encrypted messages to be read by unintended recipients, compounding the possible breaches of WhatsApp data. Ethical considerations relating to confidentiality and anonymity of human participants are thus central when collecting data via WhatsApp. This issue is especially pertinent when working with individuals in potentially precarious positions (Barbosa and Milan, 2019), as is often the case, for example, with migrant and mobile communities, who may not hold the documents required to be in a country legally.

## Scoping review methodology

The purpose of a scoping review is to identify, retrieve and synthesize literature relevant to a particular topic for the purpose of assessing the main concepts underscoring a research area and the key sources and types of available evidence (Weeks and Strudsholm, 2008b). This scoping review thus endeavours to provide not only a clearer picture of the ways in which WhatsApp is currently being used for health research but also of the opportunities and challenges that the MIM service creates.

The main stages of this scoping review were: (1) searching for relevant studies; (2) selecting studies based on pre-defined inclusion and exclusion criteria; (3) extracting data and (4) characterizing, summarizing and reporting the results. However, this process was iterative, incorporating flexibility in the movement between stages and with some repetition of steps as required to ensure a comprehensive review of the literature (Weeks and Strudsholm, 2008b).

Scoping review methodology observes many of the same steps as systematic reviews: the use of rigorous and transparent methods for data collection, analysis and synthesis remains crucial to enhance the reliability of results and the potential for replication (Weeks and Strudsholm, 2008b; Pham *et al.*, 2014; Munn *et al.*, 2018). A key difference between scoping and systematic reviews, however, is that whilst the study design as well as study findings are important considerations for both, scoping reviews do not typically include a process of quality assessment (Weeks and Strudsholm, 2008a; Grant and Booth, 2009). Thus, we did not use study quality as a criterion for selecting studies for the review.

### Search strategy

Two study investigators (K.M. and T.d.G.) simultaneously conducted a search of article titles and abstracts in five key public health electronic databases—Scopus, PubMed, SAGE Journals Online, ScienceDirect and JSTOR. The keywords ‘WhatsApp’ and ‘health research’ were combined using the Boolean operator ‘AND’, limiting the publication date from 2009 (the year when WhatsApp was first launched) to November 2019 (the time at which the search was undertaken). Sixty-nine articles were identified through the search—see Table 1 for an overview of the results. We searched both titles and abstracts, as searching and screening titles alone might miss studies using WhatsApp for data collection that did not reflect on this in the study title. Due to time and cost considerations, we limited our search to English language publications.

**Table 1:** Search strategy

Database searched, date searched	Search terms/fields	Number of references retrieved
Scopus, 18.11.2019	whatsapp (Article title, Abstract, Keywords) ‘AND’ health research (Article title, Abstract, Keywords) Limit: 2009–present	67
PubMed, 20.11. 2019	whatsapp (Title/Abstract) ’AND’ health research (Title/Abstract, Keywords) Date—Publication: 2009–present	1
SAGE, 20.11.2019	whatsapp (Abstract) ‘AND’ health research (Abstract) Publication Date: 2009–19	6
Science Direct, 20.11. 2019	whatsapp AND health research (Title, abstract or author-specified keywords) Year(s): 2009–19	5
JSTOR, 20.11.2019	whatsapp (Abstract) ‘AND’ health research (Abstract) Publication date: from 2009 to 2019	0
	Total references retrieved	79
	Duplicates	10
	Total references scanned (Abstracts)	69

### Study selection

We used the inclusion/exclusion criteria outlined in Table 2 to assign a value of ‘include’, ‘exclude’ or ‘maybe’ to the 69 identified articles in order to ascertain whether the article should be included in the review. In cases where it was not possible to decide based on the abstract alone, the full article was reviewed. Inter-rater reliability of the study selection was high with only five discrepancies, representing 6.3% of the total selected studies. Each discrepancy was a case of one reviewer coding an article as ‘maybe’ with the other coding it as ‘include’ or ‘exclude’. In all cases, the full article was retrieved and read by both investigators (K.M. and T.d.G.) to resolve the discrepancy. Following the full-paper review and exclusion of 5 additional articles, 16 articles were included in the subsequent analysis.

**Table 2:** Inclusion/exclusion criteria for selecting studies for review

Inclusion criteria	Exclusion criteria
Studies published from 2009 to 2019	Studies published outside 2009–19
English language publications	Non-English language publications
Mention of WhatsApp for health research in study abstract	No mention of WhatsApp for health research in study abstract
Original research studies	Secondary research studies

	Amount		Focus	Nature					
				Study design			Opportunities, challenges and limitations provided by WhatsApp	Impact of WhatsApp	Ethical considerations
**Article**	**Year published**	**Setting**	**Perspective**	**Discipline**	**Overall design**	**Statement on the choice of WhatsApp for data collection**			
Alsohibani *et al.* (2019)	2019	HIC	General public	Public health	Quantitative WhatsApp used to send a link to a survey on Google Form.	Nothing reported	Nothing reported	Nothing reported	The study objectives were explained to each participant by stressing the importance of the data and its confidentiality. Consent to participate was received before administering the online questionnaire
Arroz *et al.* (2019)	2019	LMIC	Healthcare providers	Health systems	Qualitative WhatsApp groups used for (flash) mentoring of implementers of a UCC programme. Used and analysed WhatsApp (written) text messages and images.	‘The choice of WA was based on the central team perception of being commonly used among health professionals in Mozambique, and with its group communication feature, interaction is perceived as being practical’.	Information sharing; clarification of administrative and financial processes reported as opportunities. Perception that as a result of the use of WhatsApp, face to face interaction was not prioritized.	Useful tool to foster central-level communication and provide implementation support. Improved efficiency of decision-making and coordination, through better and faster communication.	Researchers were participants in the groups—acted in the first instance as mentors, provided participant perspective.
Bayona *et al.* (2017)	2017	LMIC	Healthcare users	Health promotion/services	Qualitative WhatsApp used, in conjunction with other platforms, to initiate bidirectional messaging between healthcare providers and patients. Used and analysed WhatsApp (written) text messages.	Nothing specific beyond mentioning the acceptability of mHealth interventions in MSM group.	Participants were engaged in their own care, they raised questions and they elicited and managed their interpersonal support to bolster their well-being. One-fifth of the population has no mobile phone access whatsoever.	Bidirectional messaging has real potential for getting patients more actively involved in their care. Gained important insights from patient perspective that are not evident in the provider-defined care model.	Nothing reported
Fardousi *et al.* (2019)	2019	LMIC	Health care providers *paper refers to providers and users, however only research with providers was discussed	Health systems	Qualitative WhatsApp used to conduct semi-structured interviews. Used and analysed WhatsApp calls (does not specify whether voice or video calls—suspect voice calls due to issues around anonymity).	‘We used WhatsApp or Skype call functions as face-to-face and phone interviews were prohibitively difficult in opposition-controlled areas’.	Allowed for research to be conducted with a group that the researchers could not have interviewed face-to-face. All participants were men—concern that this may have been due to researchers' positionality (interviewers were male Syrian health professionals) and concerns about confidentiality. Participants from Ghouta were under-represented due to confidentiality concerns and internet availability. Reporting bias—Syrian interviews could have introduced social desirability and cultural familiarity biases (motivation-to-stay responses) and assumptions about interviewer neutrality.	Research conducted with an otherwise ‘hidden’ population. Participants indicated that they hoped that participation would raise awareness of their situation with the global community. Research could help others similarly situated to mitigate health systems challenges.	Participants were suspicious of interviews—many questioned their nature, the organization the interviewers belonged to and whether the interviewers were journalists. Participants used mobile phones to photograph and send signed consent forms; interviews were recorded anonymously using ID codes; interviewers did not ask for participant names.
Gesser-Edelsburg *et al.* (2019)	2019	HIC	Healthcare providers and the general public	Public health	Quantitative WhatsApp, among other platforms, used to distribute a survey.	Nothing reported	Noted that fewer young men responded to the survey, even though responses were carefully monitored and additional responses from specific groups were sought as deemed necessary.	Nothing reported	Nothing reported
Hazzam and Lahrech (2018)	2018	HIC	Healthcare providers	Health systems	Quantitative WhatsApp, among other platforms, used to distribute a survey.	Nothing reported	Participation limited to those using social media platforms.	Nothing reported	Nothing reported
Henry *et al.* (2016)	2016	LMIC	Healthcare providers	Health systems	Qualitative Use of a WhatsApp group as an intervention to facilitate communication. Semi-structured interviews conducted. Analysed WhatsApp text messages (excluded photos, icons, video and audio, acknowledging that photos were a key component of communication).	‘Our choice of WhatsApp reflected existing pat- terns of technology use in Kenya, where an estimated 49% of mobile phone users use WhatsApp as their preferred mobile messaging tool’.	Learning and support; planning and information sharing; improved supervision and communication; team building and moral support listed as opportunities provided by the use of WhatsApp. Considerable variation in the number of posts contributed by each participant. Noted that this may be as a result of personal preferences or power dynamics related to factors such as gender, age, or position in the community. However, this was not discussed.	With minimal training, CHWs and their supervisors tailored multi-way communication features of WA as part of enacting virtual one-to-one, group, and peer-to-peer forms of supervision and they switched channels of communication (voice calls, voice notes, photos) depending on the supervisory objectives.	Care was taken to ensure that all participants understood that they were acting as volunteers and were not obligated to participate in the project. The WhatsApp m-learning group was a closed chat group, and the project study manager moderated strict access. All WhatsApp users were instructed to obtain verbal consent before posting photos of individuals. Prior to conducting the analysis for this paper, personal identifiers were removed from chat logs, and results presented cannot be attributed to any individual participant.
Khalid *et al.* (2019)	2019	LMIC	Healthcare providers	Clinical pharmacy	Quantitative WhatsApp, among other platforms, used to disseminate a link to a survey.	Not WA specific, but is noted that online tools have been underutilized to date in data collection.	Challenges included respondents filling out the survey more than once—changes had to be made to stop this; a low response rate; and the exclusion of participants not active on social media. In addition, close to 80% of respondents were men. But no discussion of this was presented.	Nothing reported	Online questionnaire conveyed study information and statement that participation is exclusively voluntary.
Madziyire *et al.* (2017)	2017	LMIC	Healthcare providers	Clinical medicine	Quantitative WhatsApp, among other platforms, used to disseminate a link to a survey.	Nothing reported	Noted that due to the online nature of the survey, participants were unable to seek clarity on questions.	Nothing reported	Nothing reported
Pimmer *et al.* (2017)	2017	LMIC	Healthcare providers	Health systems	Mixed methods WhatsApp groups created as an intervention to provide support. Content analysed both thematically and statistically. Used and analysed (written) text messages.	Specific interest in understanding the potential of WhatsApp groups.	Information sharing and practical discussions/problem solving; the sharing of ‘lessons learnt’, performance data and acknowledgements; opportunity for management soft skills to be realized—connectedness, empathy; and usability of the app all listed as opportunities. Technological competency/mobile literacy; issues regarding power and connectivity; delayed responses; striking a balance between work and social conversations; sharing of inappropriate content; concerns around how WhatsApp inhibits ‘continuity and depth of discussion’ listed as challenges.Noted that digital technology worsens bad supervision effects. Statistical analysis of engagement did not point to any gender discrepancies.	Results in manageability and connectedness of remote distributed workforce. Flexible and dynamic form of participatory communication observed in this study can be seen as valuable ‘add-ons’ to more structured and pre-defined mobile health data collecting systems.	Training session on how to protect client privacy: WhatsApp analysis revealed no breaches of clients’ privacy and no discriminatory material was shared. To ensure equitable access for all health workers, facilitators offered individual and discreet support to health workers with restricted levels of contribution.
Raiman *et al.* (2017)	2017	HIC	Health care providers—students	Medical education	Qualitative WhatsApp groups created for medical students doing clinical work. Interviews held about students' experiences. Analysed text messages (582), images (22) and webpage links (19).	Familiarity with and popularity of the app noted, low cost, end-to-end encryption in addition to the successful use of WhatsApp in other educational and clinical settings.	Opportunities noted included educational, organizational and social opportunities. However, the lack of face-to-face interaction and difficulties in being abel to identify when students were struggling were listed as challenges.	Ease of use; improved understanding, learning and communication; provides record of discussions.	Discussion of intrusiveness and sharing of personal cell phone numbers—but noted that these were not concerns raised by participants. Discussion that WhatsApp has end-to-end encryption, unlike other platforms, providing secure platform to discuss patients.
Rasidi and Jain (2017)	2017	LMIC	Healthcare providers	Clinical dentistry	Quantitative WhatsApp used to disseminate a link to a survey.	Nothing reported	Nothing reported	Nothing reported	Nothing reported
Rathbone *et al.* (2020)	2020 *first published online in 2019	HIC	Healthcare providers	Health services	Qualitative WhatsApp group created as a means of improving team communication. Used and analysed WhatsApp text messages.	Nothing reported	Improved professional development—experiences of learning from information shared; efficient communication; and improved professional relationships all noted as opportunities. Concerns that junior level staff might come to rely heavily on support via WhatsApp and will not develop own robust problem-solving skills; delayed responses; embarrassment or fear of being judged; sharing of non-work-related messages; simplification of responses to complex clinical questions; and associated difficulties with maintaining a work-life balance all noted as challenges.	Improved communication between junior and senior pharmacists, was particularly effective during the global cyber crisis.	Noted that existing frameworks around issues like confidentiality can be replicated in spaces like WhatsApp groups. Potential legal and ethical implications of sharing patient information discussed. Engagement out of work hours not compensated. Positionality of the researchers may have affected discussions.
Karim *et al.* (2019)	2019	LMIC	Healthcare providers	Clinical medicine	Quantitative WhatsApp, among other platforms, used to distribute a survey.	Nothing reported	Nothing reported	Nothing reported	Nothing reported
Shitu *et al.* (2019)	2019	LMIC	Healthcare providers	Clinical pharmacy	Quantitative WhatsApp, among other platforms, used to distribute a survey.	Nothing reported	Exclusion of participants without social media accounts noted. Younger individuals made up a high proportion of participants. Difficult to determine response rate since the number of eligible participants who received the link is unknown.	Nothing reported	Nothing reported Participation was voluntary and consent to participate was implied by completing the survey.
Tyagi *et al.* (2019)	2019	LMIC	Healthcare users	Health services	Mixed methods Home activities of rehabilitated patients with spinal cord injury were monitored via phone calls, live video chat, and WhatsApp. Patients completed spinal cord independence measure (SCIM) pre and post-intervention, which was used to analyse their functional status. Used and analysed WhatsApp video clips.	Nothing specific to WhatsApp.	Opportunity for participants to be more actively engaged in their own care. Internet connectivity was listed as a challenge.	Cost and time effective way for healthcare to be delivered for both patients and providers.	Legal implications of telehealth raised, but not responded to.

In order to be as inclusive as possible, given the small amount of evidence currently in this area, inclusion and exclusion criteria were deliberately kept broad. For example, no exclusion criteria were defined based on study design or publication type, and we did not use study quality as an inclusion criteria (Weeks and Strudsholm, 2008a).

### Data extraction

Following the selection of the articles for review, two study investigators (K.M. and J.H.) developed a standard coding template, which was discussed with all co-investigators, to extract data from each original research article. The template was designed to include a description of the amount, focus and nature (i.e. the scope) of research related to the use of WhatsApp for health research data collection, and to support the summarizing of findings. Whilst the framework was initially developed a priori, we also followed an iterative approach, further expanding on the initial framework to comprehensively cover the findings identified in the data extraction process, in line with our iterative approach (Lavallee *et al.*, 2014).

Two study investigators (K.M. and T.d.G.) independently extracted the data from each article and entered them into the coding template, developed in Excel. One additional study investigator (J.H.) extracted data from randomly selected articles as an additional cross-check of the findings. With regards to these random checks, we achieved inter-rater reliability of the descriptive data extraction process of 100% agreement.

To describe the overall quantity of research in this field over time, we recorded the year of publication of each article. To describe the focus of the research, we extracted data on the study setting and on, broadly defined, the research participants—healthcare workers or users. To describe the nature of the research, we extracted data on the disciplinary perspective underpinning the study, characterized iteratively (elaborated below) and the study design—whether quantitative, qualitative or mixed-methods, how WhatsApp was being applied to collect data and reflections on the choice of WhatsApp for data collection. In addition, if the information was available, we included the following methodological considerations of using WhatsApp: (1) how the participants interacted with the WhatsApp interface—the opportunities and challenges, and an assessment of any social stratification implications of using the application, including gender and/or socio-economic factors, such as those discussed earlier, that can shape certain groups’ access to mobile technology; (2) the impact of WhatsApp, which refers to the researchers’ evaluation of implementing WhatsApp for health research, including technical insights and (3) the ethical implications of using WhatsApp in health research.

## An iterative approach

Whilst our coding framework was developed a priori, our categories evolved, guided by the data. For example, we expanded the category of research participants to include (in addition to healthcare users and workers) the general public, which we identified as a new code in the data. Further, we distinguished ‘health systems’ from ‘health services’, although the two disciplines are often used interchangeably. In our reading of the studies reviewed, we observed clusters that either: (1) explored the perspectives of health care providers within the health system, for the purpose of health systems strengthening or (2) involved research with healthcare users, to capture aspects of service delivery in the target population. Given these distinctions, we classified the prior studies under ‘health systems’ and the latter under the ‘health services’ umbrella.

### Collating, summarizing and reporting results

**Figure 1:**
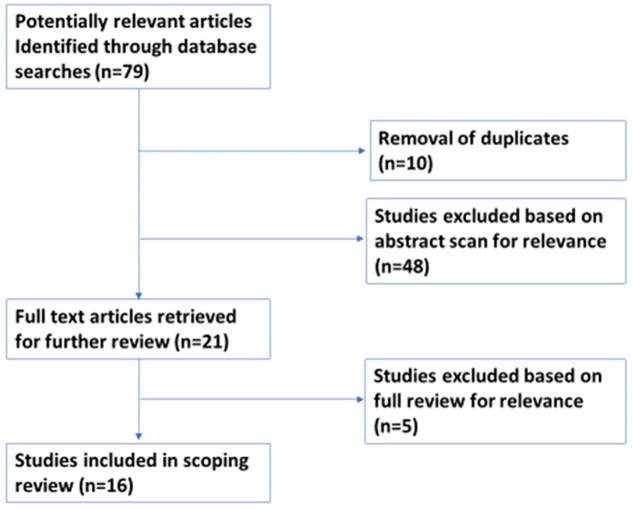
Results of search strategy and process of selecting articles on the use of WhatsApp for health research data collection.

We used a qualitative descriptive approach (Weeks and Strudsholm, 2008a) to characterize the evidence on the use of WhatsApp for health research data collection. Figure 1 summarizes the search strategy and study selection processes of the scoping review.

## Results

Our results are presented in three main categories: (1) the number of articles published per year (the amount) and focus of research; (2) discipline and study design and (3) methodological implications—a brief overview of which can be found in Table 3. As such, the first section provides a summary of the trends observed in the literature, including the number of studies published according to year, the study settings and a classification of the study subjects (health providers and/or users and/or general public). The second section distinguishes between the different disciplines that cut across the literature and the various study designs that use WhatsApp for health researchas linked to these disciplines. It further examines the study designs, including approaches to data collection and analysis, according to three classifications: (1) quantitative studies; (2) qualitative studies and (3) mixed-methods studies. In the final section, the methodological implications of using WhatsApp are elaborated according to the study designs identified in the previous section.

### Amount and focus of research employing WhatsApp as a data collection tool

We identified 16 articles that employed WhatsApp for health research in the defined time period (2009–19). All articles were published in 2016 or later, with nine articles (over half of the total) published in 2019. The articles identified covered research from a variety of contexts. Five of the studies present work undertaken in HICs; the United Kingdom (UK) (Raiman *et al.*, 2017; Rathbone *et al.*, 2020), the United Arab Emirates (UAE) (Hazzam and Lahrech, 2018), Saudi Arabia (Alsohibani *et al.*, 2019) and Israel (Gesser-Edelsburg *et al.*, 2019). The remaining 11 articles focused on research from LMICs; three present work from India (Rasidi and Varma, 2017; Karim *et al.*, 2019; Tyagi *et al.*, 2019), two from Nigeria (Khalid *et al.*, 2019; Shitu *et al.*, 2019) and one each from Kenya (Henry *et al.*, 2016); Malawi (Pimmer *et al.*, 2017); Mozambique (Arroz *et al.*, 2019); Peru (Bayona *et al.*, 2017); Syria (Fardousi *et al.*, 2019) and Zimbabwe (Madziyire *et al.*, 2017). The majority of the studies (11 out of 16) collected data on the perspective of healthcare providers, including interns (apprentices or trainees). Two studies collected data from healthcare users, one from the general public, one from the general public and healthcare providers and one from medical students.

### Nature of research employing WhatsApp as a data collection tool

#### Discipline and study design

The 16 studies identified were from a variety of disciplinary backgrounds, most commonly health systems (Henry *et al.*, 2016; Pimmer *et al.*, 2017; Hazzam and Lahrech, 2018; Arroz *et al.*, 2019; Fardousi *et al.*, 2019; Rathbone *et al.*, 2020). Additional disciplines include health services (Bayona *et al.*, 2017; Tyagi *et al.*, 2019), public health (Alsohibani *et al.*, 2019; Gesser-Edelsburg *et al.*, 2019), medical education (Raiman *et al.*, 2017) and various clinical science disciplines, including dentistry (Rasidi and Varma, 2017), medicine (Madziyire *et al.*, 2017; Karim *et al.*, 2019) and pharmacy (Khalid *et al.*, 2019; Shitu *et al.*, 2019).

Half of the studies included in the review are quantitative in nature (Madziyire *et al.*, 2017; Rasidi and Varma, 2017; Hazzam and Lahrech, 2018; Alsohibani *et al.*, 2019; Gesser-Edelsburg *et al.*, 2019; Karim *et al.*, 2019; Khalid *et al.*, 2019; Shitu *et al.*, 2019) of which the majority (*n* = 5) are from the clinical science disciplines (as listed above). None of the quantitative studies includes a statement on their decision to use WhatsApp for data collection, such as the opportunities it provides for the research in question, either generally, or compared to other online data collection approaches. In seven of the eight quantitative studies identified, WhatsApp was used—either exclusively (*n* = 2), or in combination with other social media channels (*n* = 5)—to send hyperlinks to online surveys, thereby functioning as an intermediary platform for data collection. One study (Gesser-Edelsburg *et al.*, 2019), however, used a web-based platform to build an interactive survey that was distributed via multiple social media channels, including WhatsApp. As described earlier, WhatsApp can interface with such web-based platforms that allow for the automatic administration of surveys through WhatsApp, such that participants can receive and respond to questions one at a time in the chat box. Although the above study in question does imply that the survey was administered—via several online channels—on a question-by-question basis, rather than simply distributed at one go, the authors did not elaborate on the exact process of data collection.

Across the quantitative studies, the recruitment strategies used were poorly described. Two studies (Madziyire *et al.*, 2017; Khalid *et al.*, 2019) indicate that recruitment of participants occurred before sending them the survey link via WhatsApp, without elaborating any further. In five of the studies, WhatsApp was used as the recruitment tool; authors either directly sent the survey link to pre-identified target groups, at large, as a means of recruiting potential individuals (Hazzam and Lahrech, 2018; Karim *et al.*, 2019; Shitu *et al.*, 2019); or they sent the link to a sub-set of known individuals in the target group, who then, through a snowball approach identified and forwarded the link to additional eligible participants (Alsohibani *et al.*, 2019, Gesser-Edelsburg *et al.*, 2019). The process of recruitment across these five studies, however, is vague. It appears there was no explicit strategy, and that recruitment happened passively, through simply forwarding the survey link to potential participants (and in some cases requesting them to re-forward the link further). In one study (Rasidi and Varma, 2017), there is no indication given at all as to how the participants were recruited.

Six of the studies employed a qualitative design (Henry *et al.*, 2016; Bayona *et al.*, 2017; Raiman *et al.*, 2017; Arroz *et al.*, 2019; Fardousi *et al.*, 2019; Rathbone *et al.*, 2020) and were undertaken with either a health systems or health services disciplinary focus. Of these, three studies analysed data sourced from (written) text messages sent over WhatsApp (Henry *et al.*, 2016; Bayona *et al.*, 2017; Rathbone *et al.*, 2020); one study analysed WhatsApp text messages and images (Arroz *et al.*, 2019); another one analysed text messages, images and webpage links shared via WhatsApp (Raiman *et al.*, 2017); and the final study analysed voice calls recorded over WhatsApp (Fardousi *et al.*, 2019). The data from the studies were analysed using either thematic analysis (*n* = 4) or content analysis (*n* = 2).

With one exception (Fardousi *et al.*, 2019), the qualitative studies and two mixed-methods studies (discussed below), all used WhatsApp in a tethered approach—to deliver an intervention, either for mentoring or improving access to care, with the success of the intervention subsequently evaluated through analysing the WhatsApp content that was generated as part of the intervention (as specified above and below). As exemplified in these studies, WhatsApp was used for data collection, beyond just delivering the intervention in question.

To elaborate further, two-thirds of the qualitative studies (Henry *et al.*, 2016; Raiman *et al.*, 2017; Arroz *et al.*, 2019; Rathbone *et al.*, 2020) used WhatsApp to facilitate communication between junior and senior workers for mentoring and/or educational purposes. Given the nature of these studies, since the mentoring and/or educational intervention that was delivered via WhatsApp also formed the data source, the participants in the intervention were simultaneously recruited as the subjects for the data collection component of the study.

Of these, three studies (Henry *et al.*, 2016; Raiman *et al.*, 2017; Arroz *et al.*, 2019) explicitly discuss the choice of WhatsApp for data collection, based on its popularity as a social communication tool. The other two qualitative studies included in the review employed WhatsApp (in combination with other approaches) to collect data amongst groups facing vulnerability. One study (Bayona *et al.*, 2017) describes how WhatsApp (and SMS) text messages were employed to elicit barriers and facilitators to accessing HIV health services amongst men who have sex with men (MSM) in Peru. The authors make a general observation regarding the acceptability of mHealth interventions amongst this group of individuals, without specifically justifying their choice of WhatsApp, either generally—as an instant messaging platform—or over other digital platforms. In the other study (Fardousi *et al.*, 2019), the authors describe how they selected WhatsApp (and Skype) to conduct interviews remotely, in areas where physical access was a barrier, to understand challenges experienced by healthcare providers in besieged areas in Syria. The authors indicate that they used purposive sampling to recruit healthcare providers, who were then snowballed, with each recommending two-to-three additional potential participants.

The two remaining studies included in the review employed mixed-methods approaches. Pimmer *et al.* (2017) used WhatsApp as a communication tool between healthcare workers—with a similar design and recruitment approach as the four qualitative studies described earlier—to explicitly understand its application to support healthcare work. They subsequently analysed the WhatsApp text messages, both thematically and statistically. In the other mixed-methods study (Tyagi *et al.*, 2019), rehabilitated participants with spinal cord injury sent video clips of their daily activities via email, text or WhatsApp (pre-intervention) that were then used by therapists to highlight images of wrong movements captured in these videos (as part of the intervention). The patients were recruited through a spinal rehabilitation centre. To analyse the functional status of patients pre- and post-intervention, patients completed the spinal cord independence measure (SCIM). The authors broadly infer the opportunities of telehealth to overcome barriers to continuity of care, without specific reference to the choice of WhatsApp in the study.

### Methodological implications of using WhatsApp

#### Opportunities, challenges and impact

Of the eight quantitative studies included in the review, none discuss the experiences of the research participants (positive or otherwise) while interacting with the WhatsApp interface, and neither do they evaluate the impact nor provide technical insights of implementing WhatsApp in the study. A limitation noted in three of the quantitative studies (Hazzam and Lahrech, 2018; Khalid *et al.*, 2019; Shitu *et al.*, 2019), all of which focus on health care providers, is the exclusion of participants who do not use social media platforms. Three studies, also amongst providers, describe challenges that also link to the technological nature of the research: (1) low response rates (Khalid *et al.*, 2019); (2) difficulties in determining response rates as the number of eligible participants who received the survey link were unknown (Shitu *et al.*, 2019) and (3) the inability of respondents to seek clarity on questions (Madziyire *et al.*, 2017).

With regards to the qualitative and mixed-methods study designs, the most commonly identified opportunities, as extracted from the data collected via WhatsApp (described earlier) suggest that WhatsApp is mobilized to share information (Henry *et al.*, 2016; Bayona *et al.*, 2017; Raiman *et al.*, 2017; Pimmer *et al.*, 2017; Arroz *et al.*, 2019; Rathbone *et al.*, 2020), raise questions (Henry *et al.*, 2016; Bayona *et al.*, 2017; Pimmer *et al.*, 2017; Arroz *et al.*, 2019) and support the professional development of junior-level staff (Henry *et al.*, 2016; Raiman *et al.*, 2017; Rathbone *et al.*, 2020). In addition, two studies (Pimmer *et al.*, 2017; Arroz *et al.*, 2019) cite the participatory communication function of the application as an advantage in the context of collecting group information. All the studies that used WhatsApp to facilitate communication between health professionals (Henry *et al.*, 2016; Pimmer *et al.*, 2017; Raiman *et al.*, 2017; Arroz *et al.*, 2019; Rathbone *et al.*, 2020) report improved communication as a result of using the application. Two studies (Bayona *et al.*, 2017; Tyagi *et al.*, 2019) report the usefulness of WhatsApp in overcoming barriers to continuity of care, with Bayona *et al.* (2017) further emphasizing the opportunity of employing WhatsApp as a means to provide patient perspectives that are missing in provider-defined care models. Fardousi *et al.* (2019) describe how using WhatsApp for health research in hard to access humanitarian settings can help others similarly situated to mitigate health systems challenges and raise awareness to mobilize the international community. Across several studies, authors cited the potential for discrimination or bias resulting from inadequate infrastructure, technological competency (Bayona *et al.*, 2017; Pimmer *et al.*, 2017; Arroz *et al.*, 2019, Fardousi *et al.*, 2019; Tyagi *et al.*, 2019) and gender discrepancies in access to technology (Henry *et al.*, 2016; Fardousi *et al.*, 2019) as challenges linked to using WhatsApp. Additional challenges in two studies that use WhatsApp to facilitate communication between health workers (Pimmer *et al.*, 2017; Rathbone *et al.*, 2020) point to the sharing of unrelated and/or inappropriate content, difficulties maintaining work-life balance (due to the timing of messages) and delays in responses. Several studies (Pimmer *et al.*, 2017; Raiman *et al.*, 2017; Arroz *et al.*, 2019) also point to the lack of face-to-face interaction as being problematic in the context of facilitating supervision.

#### Ethical considerations

We found little consistency between the studies with regards to efforts taken to ensure privacy, confidentiality and anonymity when using WhatsApp as a data collection tool, even in studies of a similar design.

None of the quantitative studies discussed the ethical implications of using WhatsApp for health research. Two of the studies point to some ethical measures taken to inform and protect participants in the research. Khalid *et al.* (2019) state that their online questionnaire conveyed the study information and emphasized the voluntary nature of participation. Alsohibani *et al.* (2019) cite that participants’ consent was obtained before administering the online questionnaire, but they do not elaborate on the consent process.

Across the qualitative studies, discussion of research ethics was largely missing with one notable exception. Fardousi *et al.* (2019) reported taking the following measures for obtaining informed consent and to protect the privacy of healthcare workers in besieged areas of Syria: (1) participants used mobile phones to photograph and send signed consent forms; (2) interviews were recorded anonymously using identification codes and (3) interviewers did not ask for participant names.

In four of the qualitative studies, patient data were shared between health care professionals (Henry *et al.*, 2016; Pimmer *et al.*, 2017; Raiman *et al.*, 2017; Rathbone *et al.*, 2020). However, only one of them (Pimmer *et al.*, 2017) discusses explicit training measures undertaken to prevent sharing of patient-identifying information on WhatsApp. Rathbone *et al.* (2020) highlight concerns of patient privacy, pointing to a lack of training regarding a safe way to discuss patients on the platform. On the other hand, Raiman *et al.* (2017) maintain that WhatsApp’s end-to-end encryption enables safe referral to and discussion of patients, thereby eliminating the need to anonymise the data. Similarly, Henry *et al.* (2016) indicate that the WhatsApp content that was shared between health workers was not anonymized; rather, health workers were instructed to obtain verbal consent before posting photos of patients, and personal identifiers were removed from chat logs to ensure patient confidentiality in the reporting of results. Both the study on patients with spinal cord injury (Tyagi *et al.*, 2019) and the study of health access experiences of MSM (Bayona *et al.*, 2017) report using patient data directly transmitted by the patients via the WhatsApp platform. However, neither detail how issues of patient confidentiality were handled. This finding is particularly surprising in the case of the latter, as MSM are a population group that are in many contexts marginalized and considered particularly vulnerable (Cáceres *et al.*, 2008).

## Discussion

The rapid increase in the number of studies using WhatsApp as a tool for health research published per year indicates the growing interest in this area—and reflects developments in mobile technology and the increase in WhatsApp’s user base. That most of the articles we identified describe research conducted in LMICs, with six of those in sub-Saharan Africa, is unsurprising, given that WhatsApp has particularly high penetration rates in these contexts, with India, Indonesia, Malaysia, Brazil and South Africa topping the list (Dahir, 2018; Fiesler and Hallinan, 2018). Within these studies, WhatsApp was largely used in one of two ways for health research—to send hyperlinks to online surveys, or to deliver and evaluate, either an intervention designed for healthcare users or a communication programme for healthcare providers.

Our review is limited to studies in health research databases. Using different and/or additional search terms beyond ‘health research’ (e.g. ‘health studies’ or ‘health investigations’) may have yielded more results. We reason, however, that using supplemental search terms would have produced studies similar in nature to those we identified and included in our review. Given that we observed distinct patterns across the wide range of study types and disciplines included in this review, we are confident in the interpretation of our results, including our analysis of the current (limited and nascent) state of literature using WhatsApp for health research. Indeed, the most noteworthy finding of our review is the lack of discussion on how and why WhatsApp was used by the researchers and on the potential limitations or implications of this, including, and especially with regards to ethical concerns. There is a clear need to report on these issues for digital studies, given the known challenges regarding confidentiality and data breaches. We subsequently focus on issues of research ethics in this discussion, in light of the urgent need for researchers to systematically document their use of WhatsApp and engage with its ethical issues.

In almost half of the studies we identified (*n* = 7), WhatsApp was used to facilitate data collection via online surveys. These studies offered little in the way of ethical insights for online research. In most of the surveys we located, the nature of the data collected appeared not to be sensitive, nor were vulnerable populations being surveyed. Nonetheless, the electronic and online nature of survey data add new methodological complexities surrounding data storage and security (Buchanan and Hvizdak, 2009). Given in particular that the mobile app industry is largely unregulated and cybercrime is prevalent, it would have been pertinent for authors to inform the study participants about the potential risks involved and what precautions were being taken to support the privacy and security of the participants’ data (O’Connor *et al.*, 2016).

In addition, whether individuals consider their data to be safe, secure and used appropriately by those who control it can be a key consideration in a participant’s choice to enrol in a study (O’Connor *et al.*, 2016). The perception of a survey invitation as spam or containing viruses, and the level of data security can have a possible negative impact on data quality and response rates (Scriven and Smith-Ferrier, 2003). The latter was indeed cited as an issue in several of the studies identified, without the authors providing any explanations regarding participants’ poor engagement. As we reported earlier, the recruitment approaches across the quantitative studies were poorly described and many appeared not to involve an explicit or active strategy for engaging participants. One of the main findings in a systematic review of the factors affecting engagement in digital health studies (O’Connor *et al.*, 2016) suggests that an active recruitment approach that engages with issues around privacy and security is key to overcoming barriers preventing people from participating in studies of this nature. The process of informed consent prior to the study allows researchers to establish trust with the respondents and provide an explanation of the purpose of the study, the selection criteria, how data will be employed and who will have access to it (Buchanan and Hvizdak, 2009). Obtaining informed consent and assuring that data are carefully handled is essential in academic research and imperative in digital studies (Kaufmann and Peil, 2019), given concerns with confidentiality and data breaches. However, only one of the identified survey designs cites that informed consent was obtained from the research participants. That the remaining studies failed to describe if and how they obtained participants’ consent prior to recruiting them suggests that research ethics is not foregrounded in these studies.

In the remaining half of the studies identified, WhatsApp functioned as both research field site and as a data collection tool, often involving the exchange of sensitive information. These approaches necessitate a systematic discussion of the methodological and ethical implications of the platform’s use for health research. Except for two of the studies identified (Pimmer *et al.*, 2017; Fardousi *et al.*, 2019), ethical procedures outlined were generally limited to obtaining approval from research ethics committees. With regards to digital data in qualitative research, ethical decision-making is compounded in this case by the fact that ethical review boards and respondents themselves may not understand the nuances of software-based data collection tools, including issues associated with the assumed end-to-end encryption of WhatsApp, which is often presumed to be secure (Markham and Buchanan, 2012; Boase, 2013). This resonates with data protection concerns within the mHealth field, including the observation that few African countries have comprehensive mHealth data protection legislation in place to begin with, compounding concerns about data security and privacy in LMICs (Hackett *et al.*, 2018).

The recent introduction of end-to-end encryption to WhatsApp also risks giving users a false sense of security and encourages individuals to use it also for sensitive exchanges, exposing participants to potential risks that researchers may indirectly amplify (Barbosa and Milan, 2019). In fact, the authors in one of the studies (Raiman *et al.*, 2017) explicitly discuss how the end-to-end encryption offered by WhatsApp provides a safe and secure platform to discuss patients, thereby eliminating the need to anonymize the data. However, as Kaufman and Peil (2019) explain, researchers are in fact unable to guarantee data security on the part of the platform provider as participants are also subject to WhatsApp’s terms of usage and pass over their data rights to Facebook when initially setting up their accounts. In general, we observed a lack of documentation of efforts taken, if any, to anonymize third-party data in the identified studies whereby health professionals exchanged patient data on the platform. With the exception of one of the studies (Pimmer *et al.*, 2017), the remaining four did not report any formal training on ways to safely share patient data.

Two of the studies identified in our review (Bayona *et al.*, 2017; Fardousi *et al.*, 2019) dealt with research subjects facing specific vulnerabilities that could result in serious ramifications if the data linked to them were exposed. In one study of MSM in Peru, although the authors, like others before them (Cáceres *et al.*, 2008) recognized the participants as being from a group facing marginalization and stigma in the country, they did not report taking measures to protect the subjects’ identity through anonymization of the digital data. Such measures, if taken, should be made clear in the manuscript. In the second such study, participants comprised frontline health workers in opposition-controlled areas in Syria. In this case, the authors took a systematic approach to implement full anonymisation (described earlier) in order to protect the research participants from any harm that could result from exposure of their political affiliations.

## Conclusion

The purpose of this review was to inform our approach for exploring the use of WhatsApp for data collection among migrant and mobile healthcare users in South Africa. Given our specific interest in capturing ‘real-time’ data about healthcare users’ experiences over time and place, through the administration of a survey methodology, and the unique opportunity that WhatsApp provides in this regard, we hoped to glean insights from other similar studies that may have implemented WhatsApp in this way. However, understanding the methodological opportunities, barriers and impact of using WhatsApp for health research was constrained by the limited ways in which WhatsApp has been used, and how its use has been reported, to date.

Seven out of the eight studies administering surveys used WhatsApp to send hyperlinks to online surveys, with WhatsApp functioning as a ‘static’ platform to facilitate data collection. Such use may not have warranted a discussion of the practical and logistical applications of using the software for health research. However, as described earlier, WhatsApp can also be used to administer surveys directly and ‘actively’ on the platform, an approach that appears to have been considered in one study located in our review (Gesser-Edelsburg *et al.*, 2019). The authors of this study developed a WhatsApp compatible web-based survey that has the potential to contribute to innovation regarding the nature of digital survey administration. To name a few features, these surveys can be automatically broadcasted to participants, one question at a time, with the receipt of each question being dependent on the completion of the previous one. Further, automated reminders can be sent to participants if, for example, they fail to start the survey after a certain amount of time has lapsed. Such features can enhance response rates in digital surveys, which, as cited earlier, was identified as a common challenge across several studies included in our review. The authors, however, failed to describe their method, which is a lost opportunity for future research.

Indeed with a few exceptions, most of the studies reviewed did not clearly document and describe their use of WhatsApp to collect health-related data, which makes it difficult to identify emerging best practice in this field. Given the use and acceptability of WhatsApp among hard-to-reach and often precarious communities, including asylum seekers and undocumented migrants (Kaufmann, 2018a), significant opportunities exist for the use of WhatsApp in research with these populations. However, specific methodological and ethical issues arise when working with these communities, including the uncompromising need to safeguard participant privacy (Barbosa and Milan, 2019). As such, we identify three key imperatives for researchers using WhatsApp in health research.

Primarily, given WhatsApp’s novelty as a research tool, researchers need to systematically and clearly document and discuss their use of the application when presenting their research. Current research tends to gloss over how WhatsApp is used as a research tool obfuscating understanding of best practice moving forward. Improving the state of knowledge in this regard, by documenting the challenges associated with and opportunities provided by WhatsApp, will allow for its improved use.

Secondly, given the ethical concerns regarding the use of WhatsApp, researchers must give consideration to selecting and recording only that information which is necessary to the project, encrypting the recorded data so that it is only available to the researchers, removing identifying information from the data and saving the data on secure servers (Boase, 2013). Although we do recognize that the latter recommendation poses its own challenges, as currently most universities no longer run their own servers and service, preferring to rely on commercial alternatives such as Google and Microsoft (Barbosa and Milan, 2019).

As such, when using WhatsApp as a data collection tool, researchers should endeavour to systematically and clearly document research and ethical considerations. Whereas the WHO guidelines for reporting on mHealth interventions (Agarwal *et al.*, 2016) are specific to digital programmes aimed at improving access to and use of health services—which is beyond the scope of this study—certain aspects of the guidelines are applicable to research using WhatsApp as a data collection tool. For example, the guidelines advocate the reporting of various important aspects of research design and implementation, to enhance the transparency in reporting, promote the critical assessment of digital research evidence, and improve the rigour of future reporting of research findings. In particular, item 14 of the 16-item checklist explicitly focuses on data security, entrusting researchers using mHealth to describe their data security and confidentiality protocols, including all the steps taken to secure personally identifiable information. This dimension cannot be overstated in our study, given that we have identified critical gaps in protecting the privacy and confidentiality of participant identity and health information in the current state of health research employing WhatsApp.

In addition, addressing barriers to infrastructure must be understood beyond simplified notions of the internet and/or smartphone access. As face-to-face interactions between researchers and participants are limited, additional efforts must be made to ensure that participants understand the terms of the research and are provided with information, relating to the specifics of the research project, regarding how they can seek and access support should it be required. Being able to judge whether study participants require health and/or psychological services and referring them accordingly may be difficult via WhatsApp, which raises additional ethical questions when using WhatsApp to conduct research with groups facing vulnerability. Researchers must accordingly document how they plan to overcome such challenges.

This scoping review highlights the opportunities that WhatsApp provides as a tool for health systems research, specifically with migrant and mobile communities in LMIC settings. WhatsApp is low-cost and convenient to operate, has high penetration globally, and, importantly, enables migrant and mobile users to share their location and retain their mobile phone number or WhatsApp account as they cross borders. This offers multiple opportunities for developing new approaches to health systems research in the future. However, the field of health systems research applying WhatsApp as a tool is in its infancy, and real ethical concerns exist. We urge researchers to be cognizant of the risks associated with the use of WhatsApp, to systematically document their use of the application, and to share how they address ethical challenges and concerns around data security.
